# Bilateral Looser zones or pseudofractures in the anteromedial tibia as a component of medial tibial stress syndrome in athletes

**DOI:** 10.1007/s00167-020-06290-0

**Published:** 2020-09-23

**Authors:** Julian Stürznickel, Nico Maximilian Jandl, Maximilian M. Delsmann, Emil von Vopelius, Florian Barvencik, Michael Amling, Peter Ueblacker, Tim Rolvien, Ralf Oheim

**Affiliations:** 1 Department of Osteology and Biomechanics, University Medical Center Hamburg-Eppendorf, Lottestraße 59, 22529 Hamburg, Germany; 2 Department of Orthopedics, University Medical Center Hamburg-Eppendorf, Hamburg, Germany; 3 Orthopedics and Sports Medicine, Munich, Germany

**Keywords:** Pseudofracture, Vitamin D, Looser zone, Athlete, Medial tibial stress syndrome (MTSS)

## Abstract

**Purpose:**

Medial tibial stress syndrome (MTSS) represents a common diagnosis in individuals exposed to repetitive high-stress loads affecting the lower limb, e.g., high-performance athletes. However, the diagnostic approach and therapeutic regimens are not well established.

**Methods:**

Nine patients, diagnosed as MTSS, were analyzed by a comprehensive skeletal analysis including laboratory bone turnover parameters, dual-energy X-Ray absorptiometry (DXA), and high-resolution peripheral quantitative computed tomography (HR-pQCT).

**Results:**

In 4/9 patients, bilateral pseudofractures were detected in the mid-shaft tibia. These patients had significantly lower levels of 25-hydroxycholecalciferol compared to patients with MTSS but similar levels of bone turnover parameters. Interestingly, the skeletal assessment revealed significantly higher bone mineral density (BMD) Z-scores at the hip (1.3 ± 0.6 vs. − 0.7 ± 0.5, *p* = 0.013) in patients with pseudofractures and a trend towards higher bone microarchitecture parameters measured by HR-pQCT at the distal tibia. Vitamin D supplementation restored the calcium-homeostasis in all patients. Combined with weight-bearing as tolerated, pseudofractures healed in all patients and return to competition was achieved.

**Conclusion:**

In conclusion, deficient vitamin D levels may lead to pseudofractures due to localized deterioration of mineralization, representing a pivotal component of MTSS in athletes with increased repetitive mechanical loading of the lower limbs. Moreover, the manifestation of pseudofractures is not a consequence of an altered BMD nor microarchitecture but appears in patients with exercise-induced BMD increase in combination with reduced 25-OH-D levels. The screening of MTSS patients for pseudofractures is crucial for the initiation of an appropriate treatment such as vitamin D supplementation to prevent a prolonged course of healing or recurrence.

**Level of evidence:**

III.

**Electronic supplementary material:**

The online version of this article (10.1007/s00167-020-06290-0) contains supplementary material, which is available to authorized users.

## Introduction

Medial tibial stress syndrome (MTSS) or shin splints are stress-induced injuries described by diffuse (≥ 5 cm) pain of the (postero-)medial tibia [19, 32]. It is a common injury especially in athletes exposed to a repetitive load of the lower limbs [1, 19, 28] and may display osseous signal alterations in magnetic resonance imaging (MRI) [2, 9] or translucent bone structures in radiographs and/or computed tomography (CT) [10]. There are a clinical overlap and ongoing variability of terminology in stress injuries, MTSS, pseudofractures, and stress fractures. Whereas stress injuries serve as an umbrella term for all load-induced lesions, MTSS, pseudofractures, and stress fractures represent distinct entities [25].

The pathophysiology of MTSS is still under debate with recent results suggesting biomechanical factors [3] and a disbalance of local bone remodeling with subsequent failure to repair load-induced microdamage [1, 11, 31, 32]. In most cases, clinical examination provides sufficient information to diagnose MTSS but especially when symptoms are prolonged or not characteristic, more advanced lesions and differential diagnoses (i.e., pseudofractures or stress fractures) need to be evaluated in more detail by the use of conventional imaging such as MRI/CT [30].

Pseudofractures are defined as local, radiolucent cortical defects found in patients with severe osteomalacia, caused by hereditary (e.g., X-linked hypophosphatemia) [4, 12] or in patients with severe vitamin D deficiency [13] which may occur bilateral or multilocular [15]. Supplementation of vitamin D is crucial and leads to normalization of clinical symptoms in most cases [7, 13]. In the context of repetitive high impact in athletes with underlying vitamin D deficiency, pseudofractures represent an important differential diagnosis in athletes with prolonged tibial pain [27]. As the treatment consists predominantly of establishing sufficient vitamin D levels, the detection of such lesions is of paramount importance to optimize patient outcomes and sustain physical activity in the long-term.

Nine patients presented, initially diagnosed as bilateral MTSS, of whom four had additional bilateral anterior mid-shaft pseudofractures. The aim of this study was to evaluate the specific differences regarding bone density, microstructure and turnover in MTSS patients with and without pseudofractures. The assessment included biochemical analysis (*n* = 9), bone densitometry via dual-energy X-ray absorptiometry (DXA; *n* = 9), and high-resolution peripheral quantitative computer tomography (HR-pQCT; *n* = 3).

## Materials and methods

Written informed consent of the patients or the respective legal representatives was obtained in all cases. This anonymized, retrospective chart review was performed in line with the rules of the local ethics committee (Ärztekammer Hamburg, Germany) and is in accordance with the Declaration of Helsinki. Nine patients presented who were diagnosed as bilateral MTSS but resistant to therapy. All had a history of intense and competitive physical activity. We examined these patients in our specialized outpatient clinic for musculoskeletal disorders. Diagnosis of additional pseudofractures was established by radiographs, CT and/or MRI. Patients with detected bilateral pseudofractures were compared to patients with bilateral MTSS and no (uni- or bi-lateral) pseudofractures. Body mass and height were measured in all patients before DXA measurement.

Blood samples were collected at the initial presentation to analyze markers of bone formation (bone-specific alkaline phosphatase (BAP) and osteocalcin) and bone resorption (deoxypyridinoline/crea (Dpd)). Furthermore, serum parameters of calcium metabolism (calcium, phosphate, parathyroid hormone (PTH), alkaline phosphatase (ALP), 25-hydroxycholecalciferol-levels (25-OH-D)) were assessed.

Bone mineral density (BMD) was assessed via dual-energy X-ray absorptiometry (DXA; Lunar iDXA, GE Healthcare, Madison, WI, USA) at both spine and hip. Bone microarchitecture and volumetric BMD (vBMD) was analyzed at both distal tibia and radius according to our published protocol [18] by high-resolution peripheral quantitative computer tomography (HR-pQCT; XtremeCT, Scanco Medical, Brütisellen, Switzerland) and compared to an age- and sex-matched reference values [5].

### Statistical analysis

The used software for statistical analysis was SPSS® 26 (version 26.0, IBM, Armonk, New York, USA) and GraphPad Prism® (version 7.0, GraphPad Software, La Jolla, CA). If not specified, the presented values are mean ± standard deviation (SD). Evaluation of normality of data distribution was performed by the Shapiro–Wilk test. Data of the two groups were tested for significance using Student’s *t* test for normality distributed data and Mann–Whitney *U* test for non-normally distributed data. *p* values of < 0.05 were considered as statistically significant.

## Results

**Fig. 1:**
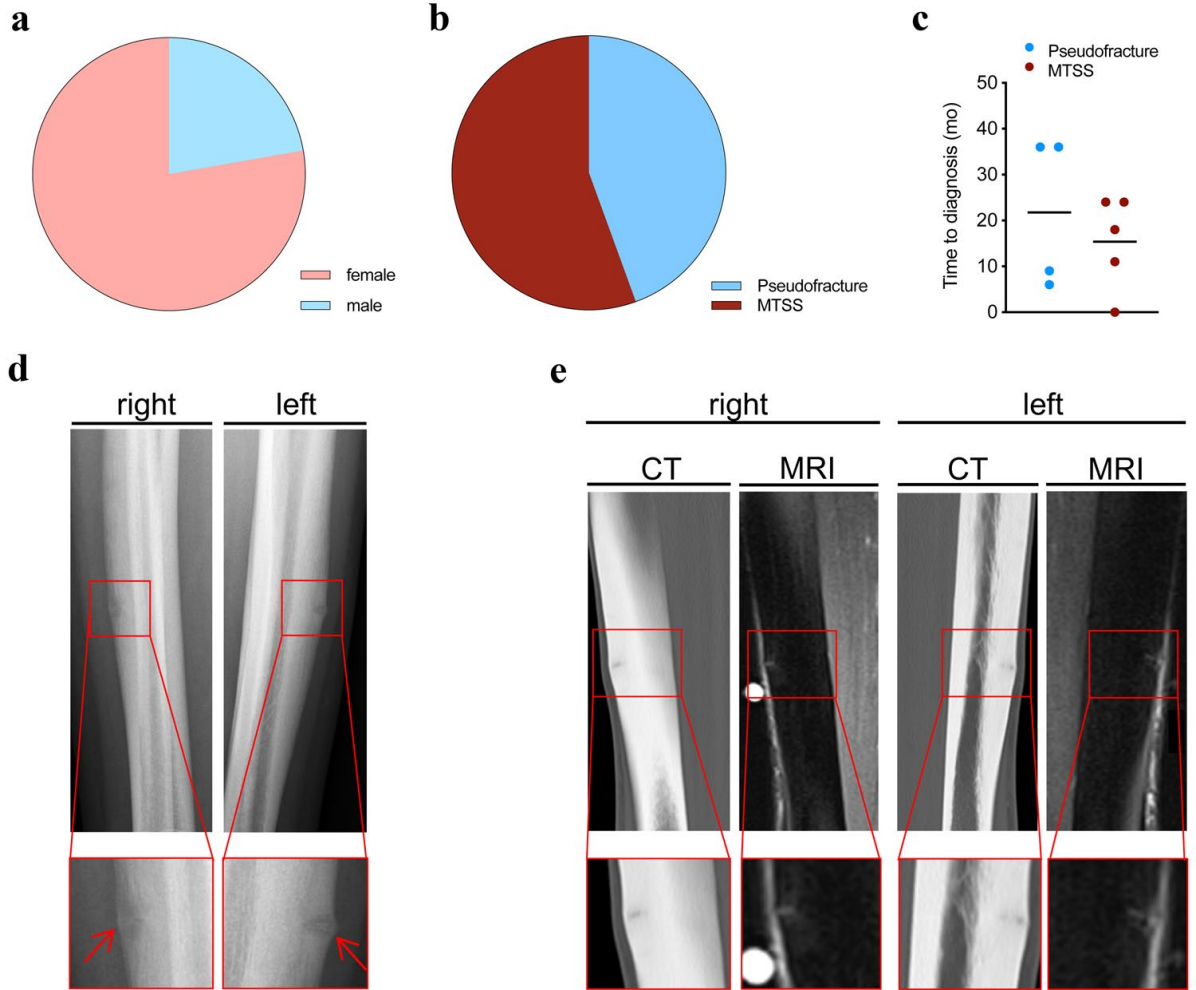
Patient characteristics and representative radiographs of bilateral pseudofractures/Looser zones at anteromedial tibiae. **a** Sex distribution of patients presenting with pain at bilateral tibiae. **b** Distribution of MTSS with and without pseudofractures in the presented patients. **c** Time from onset (in months) of clinical symptoms until diagnosis was made did not differ between the two groups. **d** Lateral view of radiographs of Patient 1 revealing bilateral Looser zones at anterior tibiae. **e** Sagittal CT and MRI images of right (left panel) and left (right panel) tibiae of Patient 4 with Looser zones at anterior tibiae

**Fig. 2:**
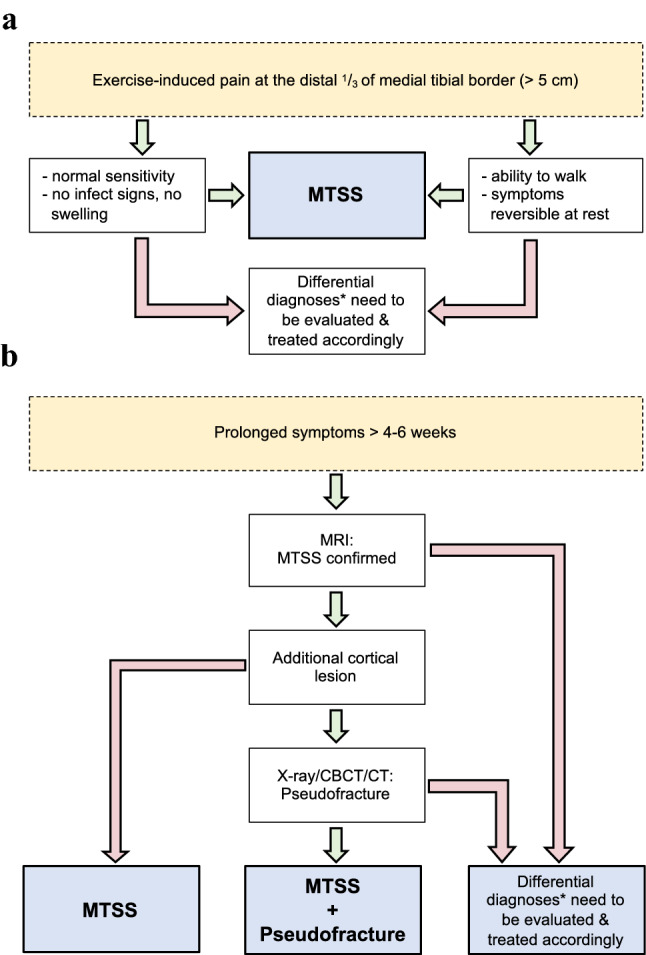
Diagnostic approach in patients with exercise-induced pain of the lower limbs. **a** Patients presenting with pain at the distal third of the tibia can be diagnosed as medial tibial stress syndrome (MTSS) if the criteria are met (green arrows). In other cases, differential diagnoses (see asterisk) should be evaluated by appropriate diagnostic approaches and treated accordingly, if applicable (red arrows). **b** In patients with suspected MTSS and prolonged symptoms despite receiving treatment, magnetic resonance imaging (MRI) scan should be obtained. After confirmation of MTSS (green arrow), MRI images should be evaluated for additional cortical lesions like pseudofractures. If results from MRI show additional (cortical) lesions not compatible to pseudofractures (e.g., stress fractures) or other signal alterations not fulfilling criteria of MTSS, underlying pathologies need to be addressed separately (red arrows). *Potential differential diagnoses include such as exertional compartment syndrome, infections (skin infections or osteomyelitis) or stress fractures. *MTSS* medial tibial stress syndrome; *MRI* magnetic resonance imaging, *CBCT* cone-beam computed tomography, *CT* computed tomography

Patient characteristics are reported in Table 1. Nine patients, mostly female (7/9, Fig. 1a) and in early adulthood (22.3 ± 7.5 years, Table 1), were included suffering from bilateral pain of the tibia. In 4/9 cases, analyzes of radiographs revealed bilateral pseudofractures of the tibia (Fig. 1b), whereas bilateral MTSS without pseudofracture was apparent in the remaining 5/9 patients. Between patients with or without pseudofractures, a trend towards higher body mass index values was revealed in those with pseudofractures. Regarding the performed sports disciplines, track and field were the most prevalent (6/9). According to patients’ reports and available files of medical history, 4/9 patients had a history of fractures. Time from onset of symptoms to diagnosis was 18.2 ± 12.8 months and did not differ significantly between groups (Fig. 1c). Detection of pseudofractures was achieved by different imaging techniques, e.g., radiographs (Fig. 1d), as well as CT and MRI (Fig. 1e), which were performed in athletes with prolonged symptoms (Fig. 2). After balancing bone metabolism, the establishment of sufficient vitamin D levels (i. e., 25-OH-D ≥ 30 µg/L), additive oral calcium supplementation (Suppl. Figure 1), non-elastic horizontal tape above the ankle distal to the muscle-to-tendon interface and transient reduction of physical activity, clinical symptoms were absent, and all patients returned to competition.

**Table 1:** Group characteristics of patients

Variable	Pseudofractures (*n* = 4)	MTSS (*n* = 5)	*p*
Sex (f/m)	3/1	4/1	–
Age (years)	21.0 ± 5.0	23.4 ± 9.6	n.s
Height (cm)	173.9 ± 2.2	172.9 ± 4.4	n.s
Weight (kg)	79.4 ± 9.3	60.1 ± 5.1	n.s
BMI (kg/m^2^)	26.2 ± 2.9	20.0 ± 0.9	n.s
Time to diagnosis (months)	21.8 ± 8.3	15.4 ± 5.4	n.s
History of fractures	1/4	3/5	–
Vitamin D (µg/L)	20.4 ± 12.4	40.6 ± 6.9	**0.017**
Z-score spine	0.4 ± 1.2	− 0.7 ± 1.8	n.s
Z-score hip	1.3 ± 0.6	− 0.7 ± 0.5	**0.013**

Individuals with MTSS and pseudofractures (Pseudofractures) and MTSS without pseudofractures (MTSS) were compared according to sex, age, morphometrics (height, weight, BMI), time from onset of clinical symptoms until diagnosis, history of fractures, and vitamin D levels at baseline. Significant values defined as *p* < 0.05 indicated in bold

*MTSS* medial tibial stress syndrome, *f* female, *m* male, *BMI* body mass index

Biochemical analysis of patients revealed significantly reduced levels of 25-OH-D in patients with pseudofractures compared to those with MTSS and no pseudofractures (Table 1). There was no significant difference in bone formation (BAP and osteocalcin) or of bone resorption markers between the two patient groups. Interestingly, 50% of the patients with pseudofractures had values above the reference range, indicating an increased bone resorption.

**Fig. 3:**
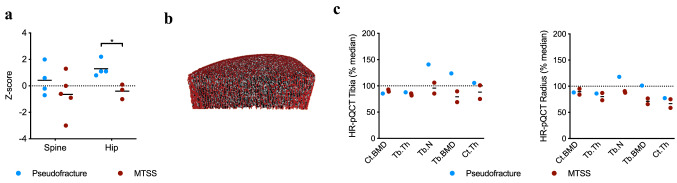
Skeletal assessment of MTSS patients presenting with or without pseudofractures. **a** Assessment of bone mineral density (BMD) via dual-energy X-ray (DXA) at both spine and hip. Interestingly, patients with pseudofractures had no impairment of BMD but significantly higher Z-scores at the hip compared to patients missing pseudofractures. **b** Representative image of high-resolution peripheral quantitative CT (HR-pQCT) analysis of distal tibia of Patient 4. **c** Analysis of bone microarchitecture at both tibia and radius via HR-pQCT in Patients 4, 9 and 10, revealing higher values in 4/5 parameters in the patient with pseudofractures compared to MTSS. Values are given as percent of the reference median [5]

**Fig. 4:**
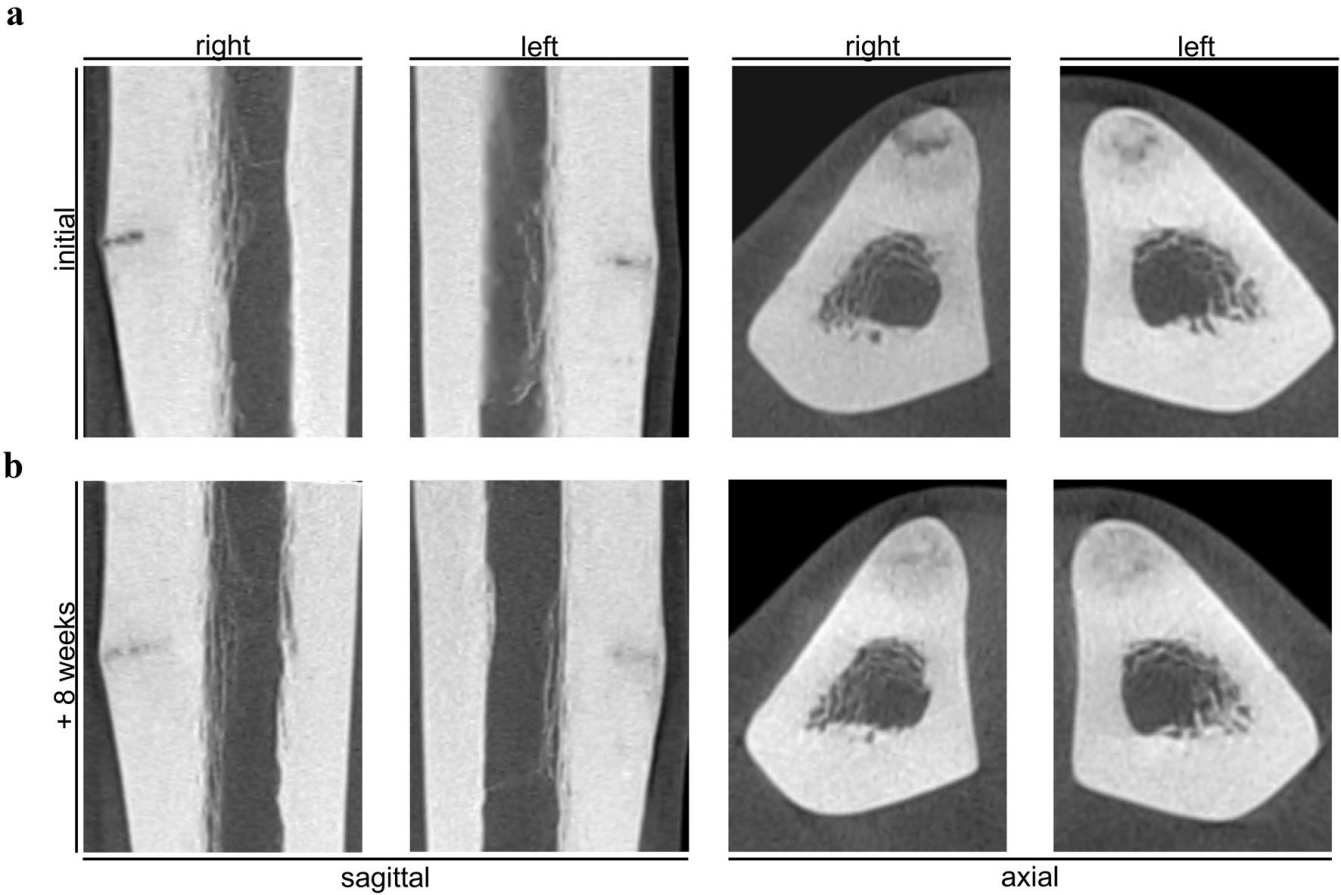
Course of lesion healing in Patient 4 assessed by cone beam computed tomography (CBCT). **a** Assessment of skeletal status at an initial presentation by CBCT revealed bilateral Looser zones at anteromedial tibiae. **b** Follow-up of radiograph after 8 weeks of intensified vitamin D supplementation (i.e., 14 days of 20,000 I.U. per day followed by 20,000 I.U. per week) and oral calcium gluconate supplementation (i.e., 1000 mg per day for three months) showed nearly complete consolidation in CBCT correlating to improved clinical symptoms

Assessment of BMD via DXA in patients with pseudofractures compared to patients without revealed significantly higher Z-scores at the hip (Fig. 3b). Parameters of bone microarchitecture assessed by HR-pQCT at the distal tibia in patient 4 with pseudofractures revealed a minor decrease in cortical volumetric BMD (Ct.BMD) and trabecular thickness (Tb.Th), whereas trabecular number (Tb.N), trabecular volumetric BMD (Tb.BMD) and cortical thickness (Ct.Th) were above reference values (Fig. 3c). In contrast, two patients with MTSS and no pseudofractures had comparably higher values of Ct.BMD, but lower values of Tb.Th, Tb.N, Tb.BMD as well as Ct.Th (Fig. 3c). Similar patterns were observed at the distal radius (Fig. 3d). Furthermore, we performed cone-beam computed tomography (CBCT) in this patient to evaluate the lesion status, revealing a nearly completed consolidation after 8 weeks of intensified vitamin D supplementation and oral calcium gluconate supplementation, correlating to improved clinical symptoms (Fig. 4).

## Discussion

The most important finding of the present study was that bilateral pseudofractures pose a relevant component or comorbidity of MTSS in athletes with deficient vitamin D levels and (physiologically) increased BMD which is clinically relevant and should not be missed. It was further demonstrated that pseudofractures are not the result of systemically attenuated skeletal status. Of note, it was revealed by our skeletal assessment that BMD and bone microarchitecture parameters were even higher in patients with pseudofractures compared to those of patients missing pseudofractures. Based on these collective findings, we stress the pivotal role of sufficient vitamin D supplementation in athletes to prevent the development of or to improve the healing of pseudofractures of the tibia.

In line with previous studies, female athletes represented the majority of our study cohort [1, 20, 32] and the time from the initial onset of clinical symptoms until diagnosis was prolonged [9, 30]. Furthermore, patients with pseudofractures showed a trend towards higher BMI values, resulting in a greater mechanical impact on the anteromedial tibia and facilitating the development of pseudofractures. This effect has, to the best of our knowledge, not been described in patients suffering from pseudofractures, but was previously reported as a relevant factor for MTSS [20].

The underlying pathomechanism of pseudofractures is an insufficient mineralization of stress-induced microdamage due to osteomalacia [15, 22]. Though available histological studies of pseudofractures and MTSS are scarce and of limited quality, described characteristics of these biopsies underline an increase in remodeling with osteoid seams [11] and especially no complete fracture in these lesions [26].

Patients with diagnosed pseudofractures had significantly lower levels of vitamin D compared to patients with MTSS and no pseudofractures, posing a risk factor for the development of insufficient mineralization with subsequent osteomalacia [14, 21] as well as an increased risk for the development of pseudofractures [7, 13, 27] and MTSS [24]. Moreover, insufficient vitamin D levels favor the development of stress injuries [29], stress fractures [23], as well as fractures in general [6].

Assessment of BMD revealed no systemic reduction but significantly higher values in patients with pseudofractures compared to those with MTSS without pseudofractures. To our best knowledge, this finding has not been stated before and indicates that systemically intact bone status does not protect individuals from the development of local bone lesions, i.e., pseudofractures or MTSS. Furthermore, elevated BMD as a physiological response to increased mechanical load may even increase the risk for the development of pseudofractures in case of simultaneous vitamin D deficiency due to higher demand for mineral supply within the context of increased bone remodeling.

Previous studies of patients with MTSS have reported locally reduced BMD in affected tibiae [17], indicating increased remodeling taking place at these sites, as DXA measures mineralized tissue only. Consequently, the skeleton is more vulnerable to the development of pseudofractures in states of chronic vitamin D deficiency. Importantly, after normalization of clinical symptoms, BMD increased again and was re-established [16].

The assessment of bone microarchitecture in the patient with pseudofractures revealed superior parameters compared to patients’ with MTSS and no pseudofracture. Supporting the results of BMD analysis, no generally impaired bone microarchitecture compared to references was detected. Interestingly, a recent study described an impairment of trabecular bone microarchitecture in soldiers affected by bone stress injuries, yet no differentiation regarding the type of lesion or comparison to reference values was conducted [25]. However, in our study, Ct.BMD was the only reduced value in the patient with pseudofracture, indicating aggravated mineralization deterioration at the cortical site correlating to the development of Looser’s zones and corresponding to the reported decrease of mineralization at the lesions’ sites [17].

In these patients, the treatment consisted of vitamin D supplementation [13, 27], additional oral calcium supplementation in cases of diagnosed pseudofractures or secondary hyperparathyroidism, and weight-bearing as tolerated followed by a gradual increase with a return to training. By applying non-elastic horizontal tape above the ankle, a redirection of forces at the tendon-to-bone insertion is achieved with greater potential potential for rehabilitation of the bone. Clinical symptoms disappeared allowing a return to competition in all patients. As this causative treatment approach is easily accessible and cost-effective, it should not be missed, especially in athletes with prolonged pain at the (anteromedial) tibia. As in general, a high rate of recurrence [32] and, in particular, tibial stress fractures [8], is known for MTSS, vitamin D supplementation should be maintained according to the individual’s needs. However, further understanding of the underlying pathomechanisms is needed to elaborate, whether higher BMD values impose an additional risk factor under simultaneous conditions of vitamin D deficiency for the development of pseudofractures and not MTSS.

## Conclusion

In conclusion, pseudofractures pose a relevant component of MTSS in athletes with prolonged pain at bilateral tibiae. The skeletal assessment revealed significantly lower values of vitamin D, significantly higher Z-scores at the hip as well as superior microarchitecture parameters in MTSS patients with pseudofractures compared to those without. The paramount importance of calcium homeostasis was highlighted, as supplementation of vitamin D and oral calcium gluconate led to the disappearance of clinical symptoms and pseudofracture consolidation. Consequently, a sufficient supplementation of vitamin D is highly recommended, especially in elite athletes, to prevent MTSS and/or pseudofractures.

## Electronic supplementary material

Below is the link to the electronic supplementary material.

**Supplemental Figure 1: **Therapeutic approach for the establishment of calcium and bone turnover homeostasis to promote healing of MTSS and/or pseudofractures. Patients with no comorbidity for hypercalcemia receive vitamin D (25-OH-D) supplementation according to their current serum levels. All patients are advised to pay attention to adequate dietary calcium intake. Furthermore, in patients with detected pseudofractures or biochemical signs of secondary hyperparathyroidism, additional calcium supplementation is prescribed for 3 months with an analysis of serum calcium levels to avoid iatrogenic hypercalcemia (PDF 14 kb)

## Abbreviations

MTSS: Medial tibial stress syndrome
DXA: Dual-energy X-Ray absorptiometry
HR-pQCT: High-resolution peripheral quantitative computed tomography
25-OH-D: 25-Hydroxycholecalciferol
BMD: Bone mineral density
MRI: Magnetic resonance imaging
CT: Computed tomography
BAP: Bone-specific alkaline phosphatase
Dpd: Deoxypyridinoline/crea
PTH: Parathyroid hormone
ALP: Alkaline phosphatase
vBMD: Volumetric bone mineral density
BMI: Body mass index
Ct.BMD: Cortical bone mineral density
Tb.Th: Trabecular thickness
Tb.N: Trabecular number
Tb.BMD: Trabecular bone mineral density
Ct.Th: Cortical thickness
CBCT: Cone beam computed tomography
