# Supplementary figures and images for: Bilateral Looser zones or pseudofractures in the anteromedial tibia as a component of medial tibial stress syndrome in athletes
Source: Knee Surg Sports Traumatol Arthrosc. 2020 Sep 23;29(5):1644–50. doi: 10.1007/s00167-020-06290-0 (PMC8038983; doi:10.1007/s00167-020-06290-0)

Suppl. Fig. 1

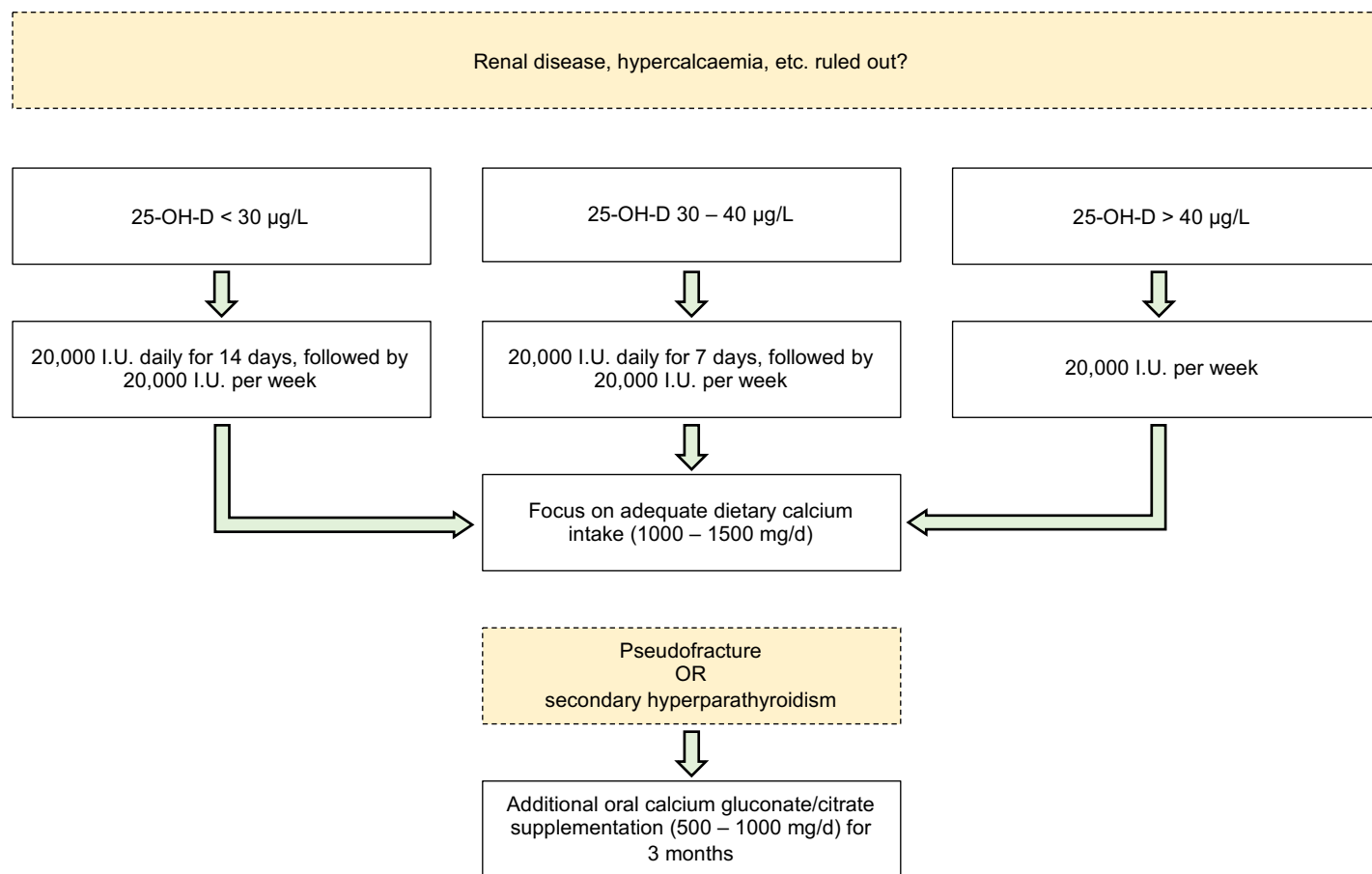

Supplement: Supplementary file 1 — Supplemental Figure 1: Therapeutic approach for the establishment of calcium and bone turnover homeostasis to promote healing of MTSS and/or pseudofractures. Patients with no comorbidity for hypercalcemia receive vitamin D (25-OH-D) supplementation according to their current serum levels. All patients are advised to pay attention to adequate dietary calcium intake. Furthermore, in patients with detected pseudofractures or biochemical signs of secondary hyperparathyroidism, additional calcium supplementation is prescribed for 3 months with an analysis of serum calcium levels to avoid iatrogenic hypercalcemia (PDF 14 kb) [file 167_2020_6290_MOESM1_ESM.pdf]
